# Current Status and Future Suggestions for Improving the Pharm. D Curriculum towards Clinical Pharmacy Practice in Pakistan

**DOI:** 10.3390/pharmacy5030046

**Published:** 2017-08-17

**Authors:** Saima Mahmood Malhi, Hassan Raza, Kiran Ajmal, Sumbul Shamim, Saniya Ata, Salman Farooq, Syed Muhammad Sharib, Sidrat-ul Muntaha

**Affiliations:** Faculty of Pharmaceutical Sciences, Dow College of Pharmacy, Dow University of Health Sciences, OJHA Campus, Karachi 74200, Pakistan; rph.hassan@hotmail.com (H.R.); kiran_ajmal@yahoo.com (K.A.); sumbul.shamim@gmail.com (S.S.); saniyaata@gmail.com (S.A.); alisalman_2008@yahoo.com (S.F.); sharib_syed@hotmail.com (S.M.S.); sidrarizwan5@hotmail.com (S.-u.-M.)

**Keywords:** Pharm. D curriculum, current pharmacy practice, patient focused program, paid residency program, questionnaire

## Abstract

**Objectives & Background**: Good curriculum is reflected as the backbone for standard universities to develop competitive professionals having great potential. Pharmacy education in Pakistan has gone through the same developmental stages as in other countries, but is still striving for improvement. In the present study, we want (i) to know the opinion on whether the current pharmacy curriculum requires any improvement in order to meet the training needs of pharmacy professionals regarding clinical knowledge and pharmacy practice; and (ii) to present some humble suggestions to decision-making authorities in order to improve it with respect to patient-focused programs (PFP). **Methods**: The study was conducted in two sessions. In first session, a questionnaire was distributed to pharmacy students of eight public/private sector universities of Karachi (*N* = 354) offering Pharm. D degrees. The second session dealt with the pharmacy teachers, deans, and practicing pharmacists in health care facilities (who are in any ways also related to academia), in order to take their opinions on and suggestions for the development of a better Pharm. D curriculum (*N* = 135). **Results**: Our results showed that 75.2% of respondents agree that the Pharm. D curriculum does not meet the international standards of practice, and 88.4% of respondents support the addition of more clinical aspects than industrial ones, as Pharm. D could be both clinically and industrially oriented, according to the needs of the Pakistani people. Furthermore, 80.2% of respondents are of the view that an apprenticeship should be included in last two years, while 88.4% demand a ‘paid residency program’ to facilitate the hospital, clinical and compounding areas of pharmacy. In addition, we also received a number of verbal suggestions for improving the Pharm. D curriculum being followed in Pakistan. **Discussion & Conclusions**: We conclude that our Pharm. D curriculum needs additions in terms of clinical practice by providing residencies and electives in health care settings. Accordingly, the need for a clinically oriented curriculum is highlighted in Pakistan, keeping in mind the continuing importance of the industrial viewpoint. Various studies have criticized the pharmacy curriculum in Pakistan in the past. Conversely, we suggest some changes in the curriculum, as change is always needed for a better tomorrow.

## 1. Introduction

A doctor of pharmacy degree program must have a multidisciplinary curriculum that produces pharmacists with sufficient mental acuity to differentiate their position as a provider of pharmaceutical care from that simply of a dispenser of drugs [1]. The Pharm. D program in the United States is the epitome of the practice-based model, as it evolved from industrial and compounding pharmacy to a more patient-focused program [2]. The pharmacy program in Pakistan was initiated as a three-year baccalaureate program and then, in 1978–1979, it was lengthened to a four-year program. At that time, the pharmacy curriculum was directed mainly towards the production of pharmaceuticals, which helped provide the pharmaceutical industry with well-qualified and skilled human resources, but there was no consideration of the public health role of the pharmacist [3]. Hence, in Pakistan, the Pharm. D degree (a 5-year program) was introduced as the basic degree in pharmacy in 2003–2004, replacing the 4-year traditional bachelor of pharmacy (B Pharm) degree [4]. Many researchers [5] have previously criticized this program, as it is just a tool to make the pharmacy students eligible for the pharmacy entrance tests of the United States and the Gulf countries. In a paper titled “Pharm. D in Pakistan: A Tag or a Degree”, the authors criticized the Pharm. D degree of being a tag, due to its lack of true clinical arrangement. Nevertheless, it is also true that the Pharm. D degree, even in United States, took years to fully develop, and it is also questionable today as to whether they have achieved their goal or not. Pakistan, being a developing country, introduced the Pharm. D degree in 2003, and a number of changes have occurred in its curriculum over the last decade. However, when this curriculum [6] was closely analyzed, it seemed that it was still in a transitional form from an industrial program to a clinical, patient-focused care program. This could be explained by the fact that the current curriculum offered to students comprises 198 credit hours in total. The yearly distribution of credit hours is as follows: first professional = 42 credit hours, second professional = 43 credit hours, third professional = 39 credit hours, fourth professional = 38 credit hours, and final professional = 36 credit hours. These are distributed across four departments; Pharmacology, Pharmaceutics, Pharmacognosy and Pharmaceutical Chemistry. Pharmacy practice has not been introduced as a fifth department at the time of this study. Clinical pharmacy courses (comprising only 6 credit hours of theory and 2 credit hours of practical work) is being taught under the supervision of department of pharmaceutics in collaboration with the department of pharmacology at this time. Thus, in order to make an effort for improvement in the curriculum, we conducted a survey to take the suggestions of ‘Pharm. D teachers and students’, so that these can be communicated effectively to the authorities for consideration.

## 2. Methodology

### 2.1. Study Design and Study Period

A cross-sectional study was conducted in two sessions by developing and distributing two different self-administered questionnaires between the months of August 2013 and March 2014. While developing the questionnaire, we tried to follow the roadmap provided by McLaughlin et al. (2013), i.e., guidelines for the conduct of educational research in terms of research design (defining, collecting, and analyzing educational data), ethical considerations, and the value of educational transformations in guiding curricular development [7]. As a practice, questionnaire-based studies in our university need not be evaluated by an institutional review board of university as a requirement; rather, they can be evaluated by getting verbal or written permission from sub-committees of the institutes under the umbrella of the university. Hence, we verbally informed the scientific review committee of the Dow College of Pharmacy about the study, and received permission following a discussion session with our dean. The survey was initially begun with pharmacy students studying in various institutes and universities of Karachi, and then subsequently continued with pharmacy teachers, deans and practicing pharmacists related to academia. The second questionnaire was developed on the basis of earlier study, addressing not only Karachi, but all of Pakistan, to obtain suggestions. 

### 2.2. Study Population and Setting

The study was conducted in a total of eight universities (either from public or private sectors) of Karachi, Pakistan, that offer Pharm. D programs; namely, Dow University of Health Sciences, University of Karachi, Federal Urdu University of Science and Technology, Jinnah Sindh Medical University, Jinnah University for Women, Baqai Medical University, Ziauddin Medical University, and Hamdard Medical University.

**First Session**-Third to fifth professional pharmacy undergraduate Pakistani students were randomly selected as the study population for the first session. A maximum of 1000 students can be enrolled in each academic year in the selected universities in total. Verbal consent was received from each student participating in the study following an explanation of the purpose of the study. Participation in the study was voluntary, and the identity of each student remained anonymous.

**Second Session**-Pharmacy teachers, deans, and practicing pharmacists in Pakistan with either four years of a B. Pharmacy or five years of a Pharm. D basic degree were randomly selected as the study population for the second session. A maximum of 250 pharmacy teachers were teaching in the selected universities at the time of study. The survey was self-addressed in all institutes in Karachi that offer a Pharm. D degree, and an online form was published to take opinions from throughout Pakistan. Teachers with basic degrees other than B. Pharmacy or Pharm. D, pharmacists working in industry, students and non-practicing pharmacists were excluded in the second session of study.

### 2.3. Data Collection

**First Session**-A 14-item questionnaire on Pharm. D in Pakistan was distributed randomly among 500 students in the first session.

**Second Session**-In the second session, a 27-items questionnaire was distributed randomly to 221 pharmacy teachers, deans, and practicing pharmacists of Pakistan at random.

All of the participants in both sessions were asked to respond to each of the items on the questionnaires, and were evaluated using a 5-point Likert scale ranging from 1 = *Strongly Disagree* to 5 = *Strongly Agree*. Any score above 3.0 was considered as positive, and below 3.0 was considered negative. 

### 2.4. Data Analysis

The retrieved questionnaires were then entered into Microsoft Excel, and then downloaded into the Statistical Package for Social Sciences (SPSS 16.0, Chicago, IL, USA) for analysis. Means and standard deviations for each of the items were calculated for both of the questionnaires.

## 3. Results

A total of 500 persons were available to respond to the questionnaire. Of these, 354 completed the questionnaire. Hence, the percentage response rate was calculated to be 70.8% for the first session. Table 1 shows the mean level of agreement of students in response to each of the 14 items in the questionnaire in the first session. Data for a total of 135 respondents were received and analyzed, out of a total of 221 professionals approached in the second session. Thus, the response rate by teachers, deans, and practicing pharmacists was estimated to be 61.1%. Table 2 shows the mean level of agreement in response to the 27-item questionnaire distributed in second session. Pharmacy deans and practicing pharmacists from all over Pakistan were included in the study population through an online survey form, and the questionnaire was self-administered in Karachi universities offering Pharm. D programs.

**Figure 1:**
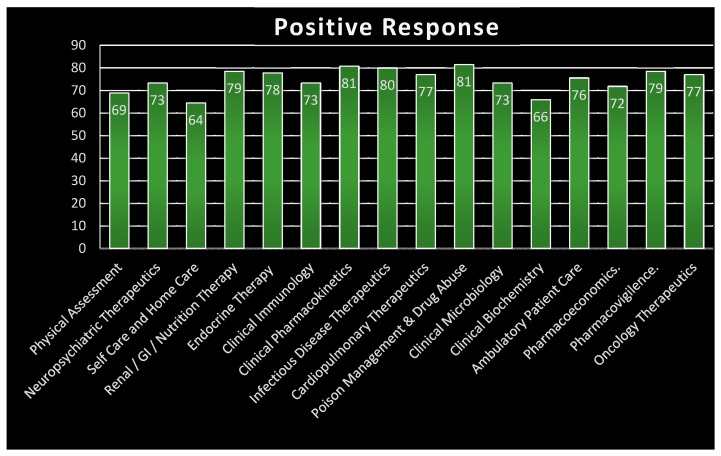
Percentage responses for various topics/electives as part of clinical pharmacy course/discipline.

Our results showed that mean level of agreement for both sessions was 4.03 and 3.93, respectively, when we analyzed the opinion that the Pharm. D curriculum does not meet the international standards of practice. For the question that Pharm. D should be both clinically and industrially oriented according to the needs of the Pakistani people, the mean level of agreement was 4.32. Regarding having a more clinically oriented or patient-focused curriculum, rather than a curriculum focusing exclusively on industrial concerns, the mean level of agreement was 4.36 and 4.33, respectively. To improve clinical knowledge and practice further, it was suggested that electives should be provided in all of the clinical disciplines (mean level of agreement for both the sessions was 4.28 and 3.99, respectively. Moreover, the mean level of agreement for starting an apprenticeship in the last two years was found to be 4.13. Last, but not least, the study revealed that a ‘paid residency program’ to facilitate the hospital, clinical and compounding areas of pharmacy should be provided to undergraduate students, and that the program should be run by a residency program director (mean level of agreement for the first session was 4.47 and for the second session was 4.37). These results are summarized in Table 1 and Table 2. Table 3 describes the percentage data of respondents regarding the division of the Pharm. D curriculum in two categories, such as pre-pharmacy and professional Pharm. D, along with the details of the subjects on which these should focus. Although most of the respondents (approx. 62% and 52%) rejected this division and the idea of pre-pharmacy, 70.3% of respondents still agreed with the suggestion of the inclusion of clinical pharmacy as a separate division, rather than as a mere course. In this connection, the percentages of responses that agreed with the idea of various topics being included as part of the clinical pharmacy course, or rather, in various courses within the discipline of clinical pharmacy, are illustrated in Figure 1).

## 4. Discussion

Alsharif, in 2012, found and reported that many pharmacy institutions worldwide were struggling with the same issues, whether in the United States or in other parts of the world, with the increased role that pharmacists play in the healthcare system on a local and global level [8]. According to economical facts and figures provided by Economic Adviser’s Wing, Finance Division, Government of Pakistan, Islamabad, Pakistan earns 20.30% of its GDP from industrial sector [9]. Pharmaceutical industry in Pakistan is the 4^th^ largest in the large-scale manufacturing sector of the country that contributes nearly 1 percent of the country’s GDP [9,10]. According to a representative of the Pakistan Pharmaceutical Manufacturers Association, (PPMA), the pharmaceutical industry in Pakistan has experienced an impressive growth of 17 per cent during 2013, which is more than the global pharmaceutical average annual growth rate of 8 per cent [9,11].

Hence, fresh pharmacy graduates have a better future in industry, compared to other “health care settings”, because clinical and community pharmacy is not truly established, even today, except in a few private hospitals. Nevertheless, current mergers of pharma industries have shifted the paradigm. Consequently, introducing a hybrid program that facilitates both of these aspects—i.e., industry and clinical/community—would be pretty good.

Previous studies related to the development of Pharm. D curricula in Pakistan have criticized it for not being productive. A few of the critical statements are as follows:

(1) The curriculum of Pharm. D Pakistan is inadequate due the lack of experienced academics [12] and practice-based facilities, and it does not contribute to health care policy [2].
(2) The Pharm. D degree was started without any planning [2].
(3) “The Pharm. D degree is just a tool to help students qualify for job opportunities in Gulf countries, as well as to make the so-called “doctors” eligible to appear for the license examination in the United States or elsewhere” [13].
(4) Whether the curriculum, without any proper clinical attachment, with just a couple of visits to hospitals, would serve the purpose is a doubtful issue [14].
(5) In Pakistan, the hospital pharmacy/clinical pharmacy is at a preliminary level [12], etc.

Instead of echoing the criticism reported in literature, we thought about the scope of the study to be discussed, i.e., that this study should not be performed to further criticize, but rather to suggest some revisions. For example:

- as the “International Pharmaceutical Federation (FIP) Global Competency Framework” suggests that pharmacy professionals must be ready in terms of their professional knowledge, skills, efficiency and availability for placements at international sites [1], we suggest that our pharmacy council should encourage the provision of a “one year paid residency program” for pharmacy students, and that the residency program should be coordinated by a residency program director (RPD), residency activities coordinators, chief resident, and/or department chair or director. In addition to the enhancement of expertise, this experience could serve to increase the students’ respect towards national, local and ethnic identities, increasing professional harmony, as well.
- Electives should be given in all disciplines of clinical pharmacy and electives should be divided into clinical and industrial categories. This will enhance their ability to integrate knowledge towards pharmacy practice.
- The pharmacy degree should be divided into a two-phase degree program, i.e., Pre-Pharmacy and Pharm. D. The Pre-Pharmacy degree should be of two or three years, and should contain all the basic subjects of formulation, and the courses needed for industry. Whereas, the Pharm. D should be more practically focused towards public health, and contain electives, an apprenticeship and internship program, and have more therapeutic subjects. As a result, depending upon the aptitude of students, the choice of degrees can be varied.
- The curriculum should place more emphasis on clinical aspects by adding therapeutics, clinical pharmacology, molecular pharmacology, clinical microbiology and pharmacogenetics as separate disciplines, rather than mere topics. In this way, students can compete internationally, having been equipped with the necessary applied knowledge following the standards.

## 5. Conclusions

Our study concludes that still, after a decade of development, our Pharm. D curriculum is facing a transition state from an industrial focus to a more patient care-oriented focus. This type of curriculum is quite beneficial for Pakistani people, and it is a necessity in this day and age. However, further improvements should be encouraged to add clinical and therapeutic courses to make this program more patient focused. For this purpose, the higher authorities should review the curriculum in the light of the suggestions of pharmacy teachers, students and, especially, practicing pharmacists given above, since our study shows more than 90% of practicing pharmacists and teachers agree to the suggested changes in the curriculum.

## Figures and Tables

**Table 1:** ‘Mean level of agreement’ of students who responded to the 14-item questionnaire for the first session on a 5-point Likert scale.

Descriptive Statistics			
Questions	*N*	Mean	Std. Deviation
1-year paid residency should be provided in a health care sector	354	4.47	0.821
The pharmacy curriculum should place more emphasis on clinical aspects	354	4.36	0.831
The pathology course is not enough for clinical practice	354	4.24	0.936
Electives should be divided into industrial and clinical categories	354	4.24	0.870
Electives should be provided in all disciplines of clinical sciences	354	4.08	0.887
Physical assessment, neuropsychiatric therapeutics, infectious disease therapeutics, poison management & drug abuse, and cardiopulmonary therapeutics should all be included as separate disciplines	354	4.08	0.966
Current pharmacy curriculum does not meet the international standards of pharmacy practice	354	4.03	0.918
Pharmacogenetics should be included as a separate course	354	3.92	0.965
The ongoing pharmacy curriculum has too many repetitions	354	3.88	0.937
The curriculum for basic medical sciences is not sufficient	354	3.86	0.902
The medical microbiology and immunological basis for therapy should be separated from pharmaceutical microbiology as a discipline	354	3.86	1.032
The discipline of pharmacognosy can be more concise, and clinical pharmacognosy can be added	354	3.79	1.004
Clinical biochemistry should be included as a separate discipline	354	3.65	1.060
I am satisfied with the changes made in the Pharm. D curriculum	354	3.46	1.072
Valid N (list wise)	354		

**Table 2:** ‘Mean level of agreement’ of teachers, deans, and practicing pharmacists who responded to the 27-item questionnaire for the second session on a 5-point Likert scale.

Descriptive Statistics			
Questions	*N*	Mean	Std. Deviation
The Pharm. D degree should include a paid residency program to facilitate hospital, clinical and compounding pharmacy	135	4.37	0.826
The Pharm. D degree should be more practical and clinically oriented	135	4.33	0.938
Pharm. D degree in Pakistan should be both clinically and industrially oriented, according to the needs of the Pakistani people	135	4.32	0.869
Elective on Poison management & drug abuse must be provided	135	4.27	0.885
The Program should be run by the residency program director (RPD), residency activities coordinator, chief resident, or department chair or director	135	4.22	0.835
Elective on clinical pharmacokinetics and laboratory data interpretation must be added	135	4.16	0.948
Elective on infectious disease therapeutics (airborne/waterborne/blood borne) must be added	135	4.16	0.979
Pharm. D should include a practical apprenticeship in last two years in different wards of tertiary health care	135	4.13	1.018
Elective on pharmacovigilance must be added	135	4.06	0.944
Elective on oncology therapeutics must be added	135	4.05	0.964
Electives on Endocrine therapy & special patient population groups (such as infants, paeds, geriatrics, pregnancy, immunocompromised and renal and hepatic insufficient patients) must be provided	135	4.05	1.002
Electives on Renal/GI/Nutrition therapy should be provided	135	4.04	0.988
Elective on cardiopulmonary therapeutics should be provided	135	4.04	0.988
Ambulatory patient care can be added as an elective	135	4.01	1.018
Electives should be provided in all the disciplines during final year.	135	3.99	1.007
Electives on pharmacogenetics and pharmacogenomics should be provided	135	3.95	0.949
Clinical pharmacy should not be included as a subject, rather it should be introduced as a discipline or department	135	3.95	1.115
The Pharm. D curriculum in Pakistan does not meet the international standards of practice	135	3.93	1.009
Elective on pharmacoeconomics should be provided	135	3.91	0.934
Clinical immunology/Hematology can be added as topics	135	3.90	0.984
Clinical microbiology can be added as topics	135	3.90	0.995
Electives on neuropsychiatric therapeutics must be provided	135	3.89	0.975
Physical assessment/Patient and family counseling can be added	135	3.85	1.062
Clinical biochemistry can be added as a subject	135	3.79	1.010
Self care and home care can also be added as subject	135	3.77	1.029
The Pre-Pharmacy includes all the basic subjects and industrial subjects necessary for a pharmacist to work in industry	135	3.33	1.112
The basic Pharm. D degree should be divided into Pre-Pharm. D and professional Pharm. D	135	3.08	1.191
Valid N (listwise)	135		

**Table 3:** Percentage responses about Pre-Pharm. D and professional Pharm. D.

The basic degree of Pharm. D should be divided into Pre-Pharm. D and Professional Pharm. D	**Valid Response**	**Frequency**	**Percent**	**Valid Percent**
	Negative Response	49	36.3	36.2963
	Neutral	34	25.2	25.18519
	Positive Response	52	38.5	38.51852
	Total	135	100	100
The Pre-Pharmacy Includes all the basic subjects and industrial subjects necessary for a pharmacist to work in industry	**Valid Response**	**Frequency**	**Percent**	**Valid Percent**
	Negative Response	37	27	27.40741
	Neutral	33	24	24.44444
	Positive Response	65	48	48.14815
	Total	135	100	100
Clinical Pharmacy should not be included as a subject rather it is introduced as a discipline or department	**Valid Response**	**Frequency**	**Percent**	**Valid Percent**
	Negative Response	22	16.2963	16.2963
	Neutral	18	13.33333	13.33333
	Positive Response	95	70.37037	70.37037
	Total	135	100	100
